# Combined aerobic and low-intensity resistance exercise training increases basal nitric oxide production and decreases arterial stiffness in healthy older adults

**DOI:** 10.3164/jcbn.19-81

**Published:** 2019-12-20

**Authors:** Takeshi Otsuki, Hayate Namatame, Toru Yoshikawa, Asako Zempo-Miyaki

**Affiliations:** 1 Faculty of Sport and Health Sciences, Ryutsu Keizai University, 120 Ryugasaki, Ibaraki 301-8555, Japan; 2 Graduate School of Sport and Health Sciences, Ryutsu Keizai University, 120 Ryugasaki, Ibaraki 301-8555, Japan

**Keywords:** nitric oxide, nitrite/nitrate, arterial stiffness, pulse wave velocity, combined aerobic and resistance exercise training

## Abstract

Meta-analyses have concluded that combined aerobic and high-intensity or moderate-intensity resistance exercise training has no effects on arterial stiffness. However, a recent study demonstrated that combined aerobic training and resistance training using rubber bands increases basal nitric oxide (NO) production and decreases arterial stiffness with marked reduction of body weight in obese adolescent girls. To investigate whether combined aerobic and low-intensity resistance training increases basal NO production and decreases arterial stiffness without body weight reduction in older adults, 27 healthy older individuals participated in a 6-week program as a part of the training group (mean body mass index, 21 kg/m^2^; walking and resistance training using one’s body weight) or the control group (22 kg/m^2^, asked not to modify their lifestyle). The exercise intervention increased aerobic capacity, muscle strength, and plasma concentrations of nitrite/nitrate (end products of NO) and decreased pulse wave velocity (an index of arterial stiffness) without changes in body weight. In the control group, there were no differences in these measures before and after the study period. These results suggest that combined aerobic and low-intensity resistance exercise training increases basal NO production and decreases arterial stiffness in healthy older adults.

## Introduction

There are two major types of exercise training: aerobic training to increase cardiopulmonary function and resistance training to strengthen skeletal muscles. Aerobic training decreases arterial stiffness,^(1–3)^ an independent risk factor for cardiovascular disease,^(4)^ possibly due to an increase in basal nitric oxide (NO) production.^(5–8)^ High-intensity resistance training increases arterial stiffness, but low-intensity resistance training seems to have no unfavorable effects on arterial stiffness.^(2,9)^ Combined aerobic and resistance exercise training is often recommended in clinical practice. Previous meta-analyses have concluded that combined training has no significant effects on arterial stiffness.^(1–3)^ However, previous studies included in these meta-analyses investigated the effects of combined aerobic and high-intensity or moderate-intensity resistance training [50–80% one-repetition maximum (1RM)] or did not describe resistance training intensity.^(1–3)^ It is possible that combined aerobic and low-intensity resistance training increases basal NO production and decreases arterial stiffness. Indeed, Wong *et al.*^(10)^ recently demonstrated that combined aerobic training and resistance training using rubber bands increases plasma concentrations of nitrite/nitrate (NOx, end products of NO) and decreases brachial–ankle pulse wave velocity (baPWV, an index of arterial stiffness) with marked reduction of body weight (−6 kg) in obese adolescent girls. In addition, combined aerobic and high-intensity resistance training [10-repetition maximum (approximately 75% 1RM)] decreased plasma NOx concentrations and did not change body weight in a study of obese adult women.^(11)^ However, it has been well known that weight loss with or without exercise training decreases arterial stiffness^(12)^ and estradiol enhances NO production.^(13)^ It is unclear whether combined aerobic and low-intensity resistance training increases basal NO production and decreases arterial stiffness when body weight does not decrease with exercise intervention or in older adults with low estradiol secretion.

We hypothesized that combined aerobic and low-intensity resistance exercise training increases basal NO production and decreases arterial stiffness even without body weight reduction in older adults. To test this hypothesis, we measured plasma NOx concentrations and baPWV before and after a 6-week program of combined aerobic training (walking) and resistance training using one’s own body weight in healthy older individuals.

## Materials and Methods

### Subjects & experimental design

Participants consisted of 12 and 15 older adults in the training and control groups, respectively. Twenty-five participants had normal body mass index (BMI <25.0 kg/m^2^), whereas a woman in the training group (BMI 25.4 kg/m^2^) and a man in the control group (27.0 kg/m^2^) were overweight. No obese persons (BMI ≥30.0 kg/m^2^) participated in this study. Participants with any disorders for which exercise is contraindicated (e.g., unstable ischemia or acute low back pain), treated or untreated hypertension (systolic/diastolic blood pressure ≥140/90 mmHg) or diabetes, taking hormone replacement therapy, or smoking tobacco were excluded. Subjects refrained from alcohol consumption and intense physical activity starting on the day before testing and caffeine consumption on the day of testing. In addition, subjects were instructed not to consume NOx from dietary sources^(14,15)^ on the day before blood sampling. Subjects were asked not to change their lifestyle during the study period except for participation in this study. Compliance with the study protocol was monitored through questions asked before measurements and blood sampling. All measurements were conducted in an air-conditioned room (air temperature, 25°C).

This study was approved by the Ethics Committee of Ryutsu Keizai University (approval number 15). The study protocol conformed to the principles of the Declaration of Helsinki. All participants provided written informed consent prior to study participation.

### Blood chemical analysis

Blood samples were collected after an overnight fast. Subjects were instructed not to take NOx from dietary sources^(14,15)^ on the day before sampling. Plasma NOx concentrations were determined using the Griess method.^(16,17)^ Lipid profile and levels of glucose, insulin, and hemoglobin A1c were determined using standard techniques.^(18)^

### baPWV, blood pressure, and heart rate measurement

Brachial and posterior tibial artery pulse waves were obtained in triplicate in the supine position (BP-203RPE II; Fukuda Colin, Tokyo, Japan).^(19)^ The device calculated the distance traveled by the pulse waves based on each subject’s height and automatically determined the pulse wave transit time. baPWV was calculated as the distance divided by the transit time. Brachial arterial blood pressure and heart rate (HR) were measured using oscillometry and electrocardiography, respectively, at the time of waveform recording (BP-203RPE II; Fukuda Colin).

### Maximal oxygen uptake estimation

Three-lead electrocardiography (LRR-03; GMS, Tokyo, Japan) and breath-by-breath oxygen uptake (AE300S; Minato Medical Science, Osaka, Japan) were monitored during incremental cycling of 4 min at 30 W with a 20 W increase for males or a 15 W increase for females every 2 min to 85% of age-predicted maximum HR.^(19)^ Maximal oxygen uptake was calculated as oxygen uptake corresponding to the age-predicted maximal HR using the linear regression line for HR and oxygen uptake.

### Sit-to-stand test

A steel molded chair was used for the 10-time sit-to-stand test.^(20)^ Participants were asked to stand up from a sitting position and then to sit down 10 times as fast as possible. They were instructed to stand up fully and to place their buttocks on the chair in a sitting position between repetitions. Time was measured using a stopwatch. Prior to the measured trial, practice trials with submaximal effort were performed for positioning and learning of the task.

### Exercise intervention

Participants in the training group underwent supervised aerobic (walking) and resistance training once per week for 6 weeks. HR was monitored during 30 min of walking using a HR monitor (RS-400; Polar, Kempele, Finland). During the first 10 min of walking, participants walked at normal speed as a warm-up.^(7)^ Ten minutes after the onset of walking, participants increased their walking speed to the target intensity. Initially, the target intensity was relatively low (60–65% of age-predicted maximal HR). As exercise tolerance improved, the intensity increased to 75% of the age-predicted maximal HR.

Resistance training, focused on the lower extremities, consisted of four exercises using one’s body weight (squats, lunges, calf raises, and hip extension exercises). Resistance training also increased in intensity (e.g., first, sitting on chair-to-standing up; second, squat; and third, squat loaded with a rubber band) and volume (increasing from 8 up to 10 repetitions and from 2 up to 3 sets) during the 6 weeks.

In addition to supervised training, participants walked and performed resistance training twice per week at home. Participants were asked to record these activities in a training log. On supervised training days, resistance training was performed before walking, but the order was arbitrarily determined by each participant on unsupervised days. Some participants walked and performed resistance training on separate days. Participants in the control group were asked not to modify their lifestyle during the study period.

### Statistical analysis

Values are presented as means ± SE. The unpaired *t* test or chi-squared test was used to detect intergroup differences in variables before the intervention, as appropriate. Effects of the intervention were tested using repeated measures two-way analysis of variance. If a significant *F* value was found in the interaction between group and time, a Fisher’s post hoc test was performed. Effect size (ES) was also calculated for outcome measures (i.e., exercise parameters, plasma NOx concentration, and baPWV) to exclude type II error due to the small sample size. If a *p* value <0.05 or ES value ≥0.5 was observed, the intergroup difference or change from before to after the study period was considered significant. ES values ≥0.50 and ≥0.80 were interpreted as medium and large ES, respectively.^(21)^ G*Power^(22)^ and StatView statistical software (SAS Institute, Cary, NC) were used for analysis.

## Results

In the training group, compliance with the exercise prescription was 110 ± 15% for walking (100% equals 30 min × 3 times/week) and 95 ± 6% for resistance exercise (100% equals 4 exercises × 8–10 repetitions × 2–3 sets × 3 times/week). There were no intergroup differences in the male-to-female ratio, age, BMI, or laboratory values before the study period (Table 1). BMI did not change during the study period in either group. Serum concentrations of low-density lipoprotein cholesterol in the training group were higher after the intervention period compared to baseline and the control group, respectively. Plasma glucose concentrations in the control group were higher after the study period relative to baseline and the training group, respectively. Plasma hemoglobin A1c levels in the training group was lower after the training period vs baseline and the control group, respectively. Maximal oxygen uptake before the study period was lower in the training vs control group (Table 2). However, maximal oxygen uptake increased with the training program, which showed no intergroup difference after the study period. There was no intergroup difference in the sit-to-stand time before the study period. Sit-to-stand time in the training group was lower after the training period vs baseline and the control group, respectively. In the control group, there were no differences before vs after the study period in these measures.

**Fig. 1:**
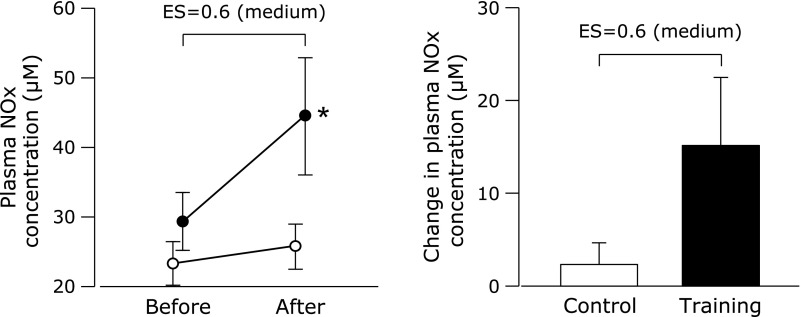
Plasma nitrite/nitrate (NOx) concentrations before and after a 6-week combined aerobic and low-intensity resistance exercise training intervention. Values are means ± SEs. Black circles, training group; white circles, control group. The *p* value of the interaction between group and time was 0.07. * Effect size (ES) = 0.8 vs the control group.

**Fig. 2:**
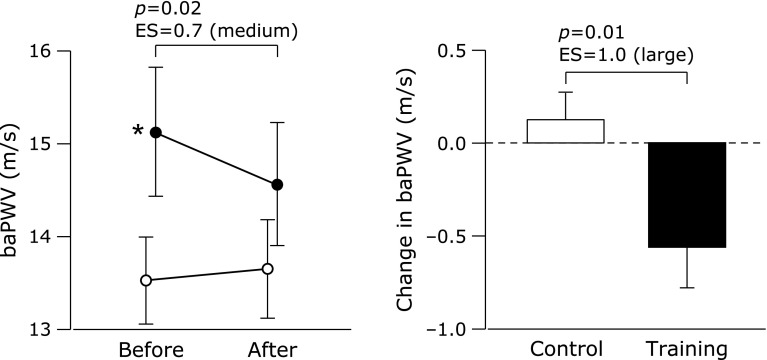
Brachial–ankle pulse wave velocity (baPWV) before and after a 6-week combined aerobic and low-intensity resistance exercise training intervention. Values are means ± SEs. Black circles, training group; white circles, control group. The *p* value of the interaction between group and time was 0.01. *Effect size (ES) = 0.7 vs the control group.

There were no intergroup differences in blood pressure or HR before the study period (Table 3). These variables did not change during the study period in either group. Similarly, there was no intergroup difference in plasma NOx concentrations before the study period (Fig. 1). The training program increased plasma NOx concentrations, whereas in the control group there was no difference before vs after the study period. After the study period, the training group had higher plasma NOx concentrations than the control group. The training group had higher baPWV before the study period but baPWV decreased with the training program, whereas baPWV did not change in the control group (Fig. 2). There was no intergroup difference in baPWV between the groups after the study period.

## Discussion

We investigated physical fitness, basal NO production, and arterial stiffness before and after a 6-week program of combined walking and resistance training using one’s body weight in healthy older adults. First, maximal oxygen uptake and sit-to-stand time improved after the training program, implying that the program had sufficient intensity and volume to improve aerobic capacity and muscular strength. Second, the training program increased plasma NOx concentrations and decreased baPWV. In the control group, there were no changes in these measures over the study period. These results suggest that combined aerobic and low-intensity resistance exercise training increases basal NO production and decreases arterial stiffness in healthy older adults.

A previous study reported that combined aerobic training and resistance training using rubber bands increased basal NO production and resulted in marked reduction of body weight in obese adolescent girls.^(10)^ This study expanded that research outcome to an intervention without body weight reduction and in healthy older adults. A meta-analysis reported that combined aerobic and resistance training increases NO bioavailability, but the included studies used flow-mediated vasodilation (FMD) as an index.^(23)^ This means that the review article did not focus on basal NO production or elastic arteries.^(23)^ FMD assesses the responsiveness of the vascular endothelium to an increase in shear stress and the endothelial function of peripheral muscular arteries. Arterial stiffness at resting is reflected by basal NO levels but not responsiveness. Stiffness is important in central elastic arteries that buffer blood pressure pulsations and generate continuous blood flow. Therefore, this study investigated basal and systemic (i.e., including elastic central arteries) markers of NO production.

This study demonstrated that combined aerobic and low-intensity resistance exercise training increases plasma NOx concentrations and decreases baPWV but did not show a correlation between changes in these measures probably due to the small sample size. This study did not include a mechanistic experiment such as administration of a NO synthase inhibitor. However, previous studies reported that increases in plasma NOx concentration with aerobic exercise training are correlated with decreases in arterial stiffness^(5,7)^ and that local or systemic administration of an NO synthesis inhibitor increases arterial stiffness.^(24,25)^ Based on these previous studies, a decrease in arterial stiffness associated with combined aerobic and low-intensity resistance training might be due to an increase in basal NO production.

Combined aerobic and resistance training decreased arterial stiffness in this study, even though previous meta-analyses have concluded that combined training has no significant effects on arterial stiffness.^(1–3)^ An important difference between this study and previous studies may be exercise intensity. High-intensity resistance training increases arterial stiffness.^(2,9)^ In most previous studies analyzed in the meta-analyses, participants performed resistance training at high or moderate (50–80% 1RM) intensity. However, some studies explained the exercise prescription (e.g., name of exercises and number of repetitions and sets) very briefly and did not describe exercise intensity.^(1–3)^ Among the studies not describing exercise intensity,^(1–3)^ only Miura *et al.*^(26)^ described the exercise prescription in detail and reported that combined aerobic and resistance training using rubber bands and light dumbbells, conceivably performed at low intensity, decreased arterial stiffness. It is difficult to compare exercise intensity between this study and previous study,^(26)^ but the intensity might have been lower in this study because a rubber band was only used during the squat and hip extension exercises by participants with increased exercise tolerance and no dumbbells were used. As the result, the combined training program in this study decreased arterial stiffness. In addition, it should be noted that walking and leg resistance exercises in this study increased leg muscle strength despite the low intensity of the resistance exercises.

Effects of exercise intervention were tested in this study, but dietary modification may enhance the effects of an exercise program on NO production, arterial stiffness, and physical fitness. For example, we previously showed that *Chlorella*-derived dietary supplementation increases basal NO production and decreases arterial stiffness.^(17,27)^ Zempo-Miyaki *et al.*^(28)^ demonstrated that adding exercise training to dietary modification enhances decreases in arterial stiffness vs dietary modification only. Nagai *et al.*^(29)^ reported that arm and trunk muscle mass in older individuals increase after a 12-week resistance-training program with maslinic acid supplementation. Ha *et al.*^(30)^ found that aerobic capacity in the 6-min walking test in older individuals increases after a 12-week aquatic exercise program with burdock extract supplementation. Kumagai *et al.*^(31)^ reported that adding higher daily physical activity levels to calorie restriction is needed in overweight and obese men for increasing circulating levels of testosterone, a hormone associating with health status and physical fitness. Taken together, dietary modification seems to enhance the effects of exercise programs on multiple aspects of health and physical fitness. On the other hand, oral glucose ingestion is suggested to increase leg arterial stiffness.^(32)^ Further studies are warranted to establish more efficient lifestyle modifications.

In conclusion, combined aerobic and low-intensity resistance exercise training increases basal NO production and decreases arterial stiffness in healthy older adults.

## Figures and Tables

**Table 1:** Characteristics of the study participants before and after a 6-week combined aerobic and low-intensity resistance exercise training intervention period

		Before	After	Interaction
*n* (male/female)	Control	7/8	—	—
	Training	4/8	—	
Age (years)	Control	64 ± 2	—	—
	Training	68 ± 2	—	
Body mass index (kg/m^2^)	Control	22 ± 1	22 ± 1	*p* = 0.49
	Training	21 ± 1	21 ± 1	
HDL cholesterol (mg/dl)	Control	74 ± 6	73 ± 5	*p* = 0.11
	Training	67 ± 4	72 ± 5	
LDL cholesterol (mg/dl)	Control	137 ± 7	135 ± 6	*p* = 0.02
	Training	152 ± 13	165 ± 12*^,^^†^	
Triglycerides (mg/dl)	Control	113 ± 11	99 ± 10	*p* = 0.26
	Training	103 ± 10	105 ± 12	
Glucose (mg/dl)	Control	93 ± 3	98 ± 2***	*p*<0.01
	Training	92 ± 1	89 ± 2^†^	
Hemoglobin A1c (%)	Control	5.5 ± 0.1	5.6 ± 0.1	*p*<0.01
	Training	5.4 ± 0.0	5.3 ± 0.0**^,^^†^	
Insulin (µU/ml)	Control	5 ± 1	6 ± 1	*p* = 0.64
	Training	4 ± 0	5 ± 1	

Values are means ± SEs. *p<0.05, **p<0.005, and ***p<0.0005 vs baseline and ^†^*p*<0.05 vs the control group. HDL, high-density lipoprotein; LDL, low-density lipoprotein.

**Table 2:** Physical fitness before and after a 6-week combined aerobic and low-intensity resistance exercise training intervention period

		Before	After	Interaction
Maximal oxygen uptake (ml/kg/min)	Control	25.6 ± 1.6	25.1 ± 1.3	*p* = 0.05
	Training	21.7 ± 1.3^§^	23.5 ± 1.6^†^	
Sit-to-stand (s/10 times)	Control	13.6 ± 0.5	13.9 ± 1.2	*p*<0.05
	Training	14.5 ± 1.0	9.0 ± 0.6*^,^^††^^,^^‡^^,^^§§^	

*p<0.0005, ^†^effect size (ES) ≥0.5, and ^††^ES≥0.8 vs baseline, ^‡^*p*<0.005, ^§^ES≥0.5, and ^§§^ES≥0.8 vs the control group.

**Table 3:** Blood pressure and heart rate before and after a 6-week combined aerobic and low-intensity resistance exercise training intervention period

		Before	After	Interaction
Systolic blood pressure (mmHg)	Control	117 ± 3	114 ± 4	*p* = 0.99
	Training	120 ± 4	117 ± 3	
Mean blood pressure (mmHg)	Control	89 ± 3	87 ± 3	*p* = 0.89
	Training	92 ± 3	89 ± 2	
Diastolic blood pressure (mmHg)	Control	70 ± 3	69 ± 3	*p* = 0.74
	Training	69 ± 2	67 ± 2	
Pulse pressure (mmHg)	Control	47 ± 1	45 ± 2	*p* = 0.76
	Training	52 ± 3	50 ± 3	
Heart rate (bpm)	Control	63 ± 2	64 ± 2	*p* = 0.55
	Training	61 ± 3	61 ± 2	

Values are means ± SEs.

## Abbreviations

baPWV: brachial-ankle pulse wave velocity
BMI: body mass index
ES: effect size
FMD: flow-mediated vasodilation
HDL: high-density lipoprotein
HR: heart rate
LDL: low-density lipoprotein
NO: nitric oxide
NOx: nitrite/nitrate
1RM: one-repetition maximum
